# Author Correction: Fine-scale heterogeneity in population density predicts wave dynamics in dengue epidemics

**DOI:** 10.1038/s41467-022-29163-1

**Published:** 2022-03-11

**Authors:** Victoria Romeo-Aznar, Laís Picinini Freitas, Oswaldo Gonçalves Cruz, Aaron A. King, Mercedes Pascual

**Affiliations:** 1 Department of Ecology and Evolution, University of Chicago, Chicago, IL USA; 2 Departamento de Ecología, Genética y Evolución, and Instituto IEGEBA (CONICET-UBA), Facultad de Ciencias Exactas y Naturales, Universidad de Buenos Aires, Ciudad Universitaria, Pabellón 2, C1428EHA Buenos Aires, Argentina; 3 Mansueto Institute for Urban Innovation, The University of Chicago, Chicago, IL USA; 4 Postgraduate Program of Epidemiology in Public Health - Escola Nacional de Saúde Pública Sergio Arouca - Fundação Oswaldo Cruz, Rio de Janeiro, Brazil; 5 Programa de Computação Científica - Fundação Oswaldo Cruz, Rio de Janeiro, Brazil; 6 Department of Ecology and Evolutionary Biology, University of Michigan, Ann Arbor, MI USA; 7 Center for the Study of Complex Systems, University of Michigan, Ann Arbor, MI USA; 8 The Santa Fe Institute, Santa Fe, NM USA

**Keywords:** Ecological epidemiology, Ecological modelling, Epidemiology, Dengue virus, Computational models

Correction to: *Nature Communications* 10.1038/s41467-022-28231-w, published online 22 February 2022.

The original Article contained two sentences in the Abstract which duplicated the same information. They read “Using surveillance data at fine resolution from Rio de Janeiro, we document a scale-invariant pattern in the size of successive epidemics following DENV4 emergence. Using surveillance data at fine resolution following the emergence of the DENV4 dengue serotype in Rio de Janeiro, we document a pattern in the size of successive epidemics that is invariant to the scale of spatial aggregation.”. The first sentence has now been removed.

This correction has been made in both the PDF and HTML versions of the Article.

