# Supplementary material for: Fine-scale heterogeneity in population density predicts wave dynamics in dengue epidemics
Source: Nat Commun. 2022 Feb 22;13:996. doi: 10.1038/s41467-022-28231-w (PMC8864019; doi:10.1038/s41467-022-28231-w)
Supplement: Supplementary file 1 — Supplementary Information [file 41467_2022_28231_MOESM1_ESM.pdf]

Fine-scale heterogeneity in population density predicts wave dynamics in dengue epidemics

Victoria Romeo-Aznar (1,2,3), Laís Picinini Freitas (4, 5), Oswaldo Gonçalves Cruz (5), Aaron A King (6,7,8) and Mercedes Pascual* (1,8).

Supplementary Information

Name	Value	Parameter
μ	1/(75*365) days^{-1}	mortality rate
γ	1/17 days^{-1}	recovery rate
β_0	1.15/17 days $^{-1}$	mean transmission rate
ω	2*π/365 days^{-1}	annual frequency of seasonality
$\phi_{stochastic}$	1.60	seasonality phase in the stochastic model
$\phi_{deterministic}$	2.0	seasonality phase in the deterministic model
$\delta_{stochastic}$	0.2	Amplitude of transmission rate in the stochastic model
$\delta_{deterministic}$	0.99	Amplitude of transmission rate in the deterministic model

Supplementary Table 1: Parameters of the *SIR* model (from (1))

Supplementary Figure 1

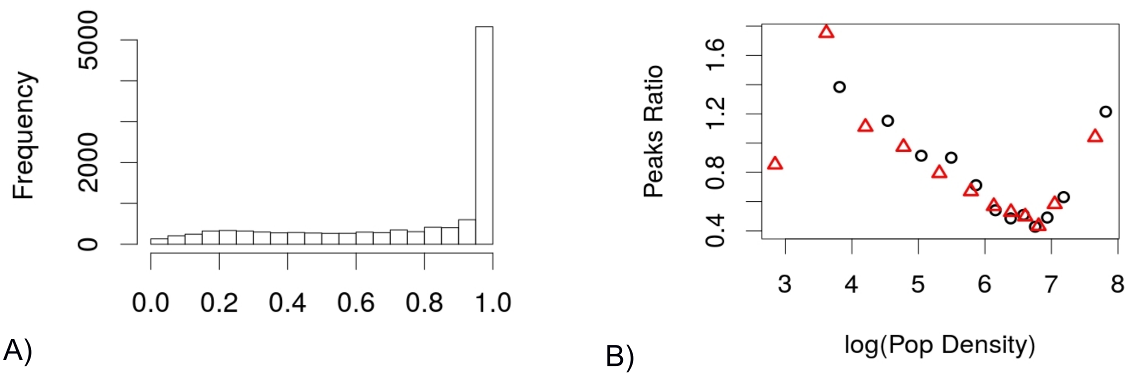

Supplementary Fig. 1. **(A)** Histogram of the ratio of unit areas computed with and without non-urban areas. **(B)** Peak ratio vs the natural logarithm of population density. Black circles correspond to population density computed as population over the effective area of the unit (i.e. without non-urban areas), and red triangles correspond to population per unit.

Supplementary Figure 2

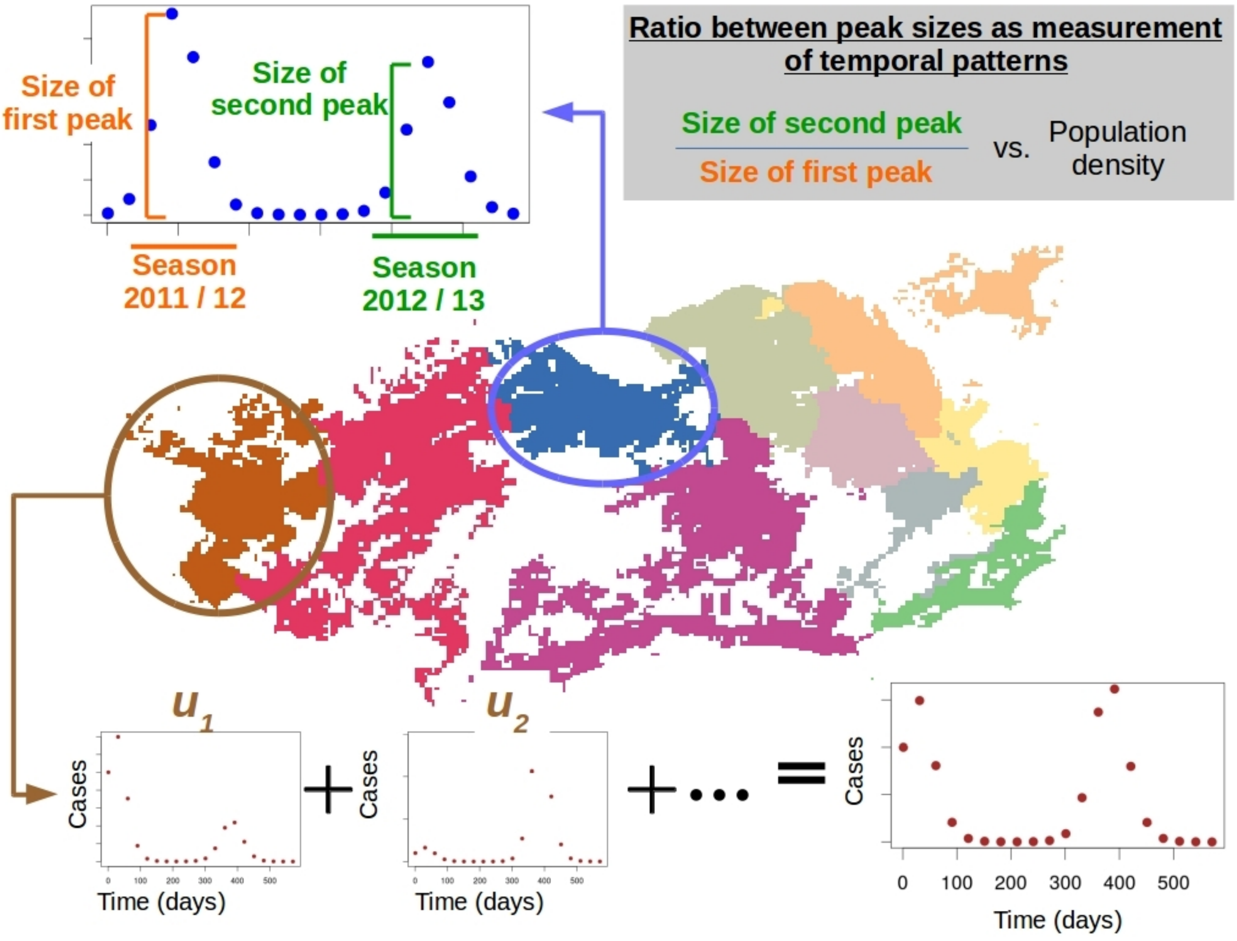

Supplementary Fig. 2. Diagram illustrating the aggregation of units when considering incidence patterns. This example considers the 10 administrative regions of Rio de Janeiro and shows the peak ratio for the resulting global time series.

Supplementary Figure 3

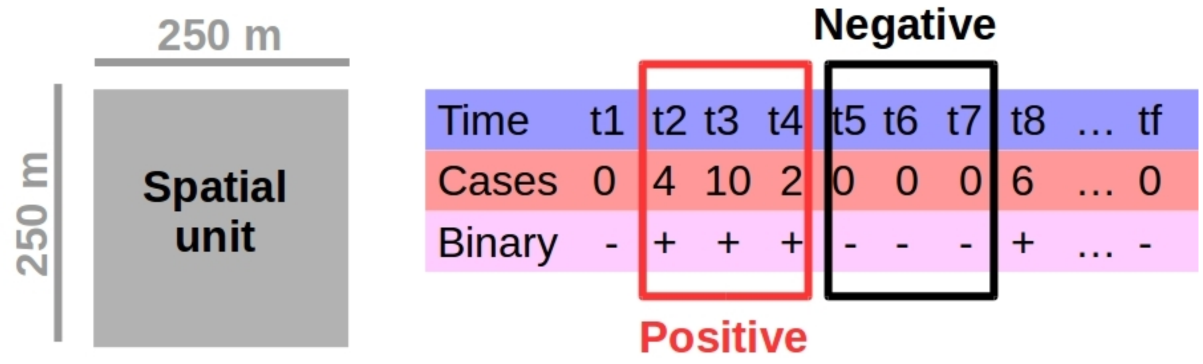

Supplementary Fig. 3: Example of a time series of reported cases, illustrating the definition of negative and positive states for the spatial unit**.**

Supplementary Figure 4

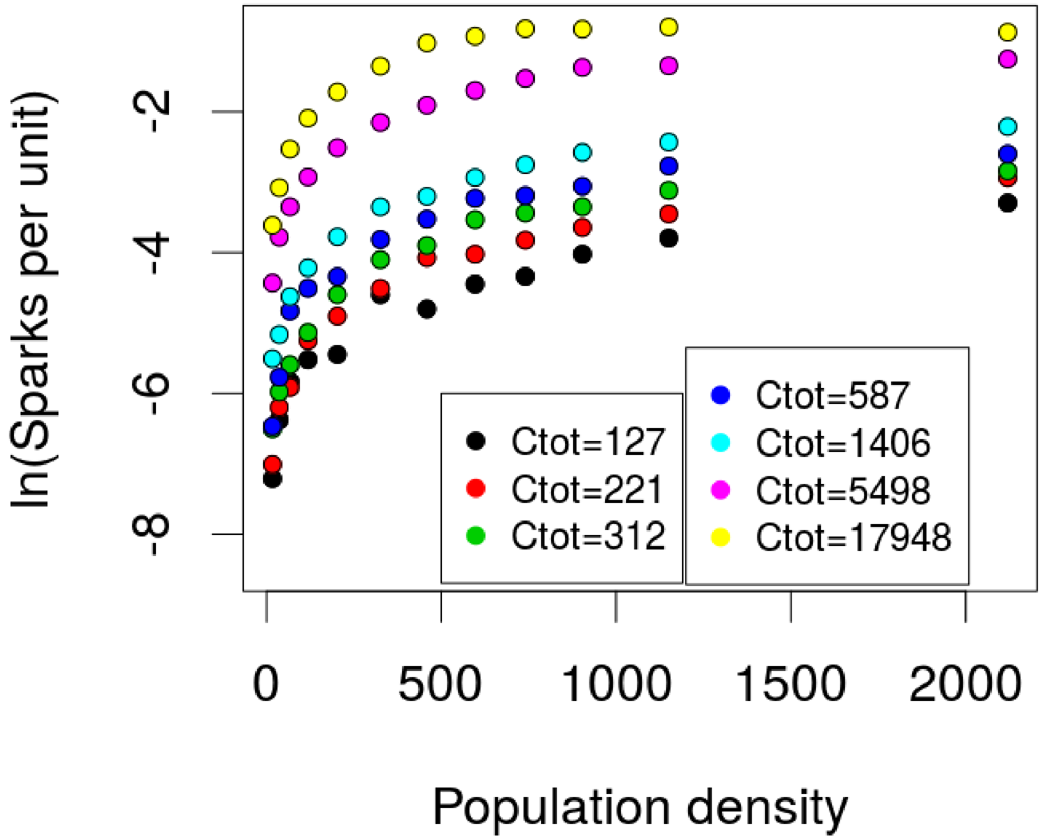

Supplementary Fig. 4: Natural logarithm of the number of dengue sparks per spatial unit estimated from the reported data as a function of the unit's mean population density in Rio de Janeiro city. The colors represent a different total number of cases in the city. As C_{Tot} increases, more sparks are produced.

Supplementary Figure 5

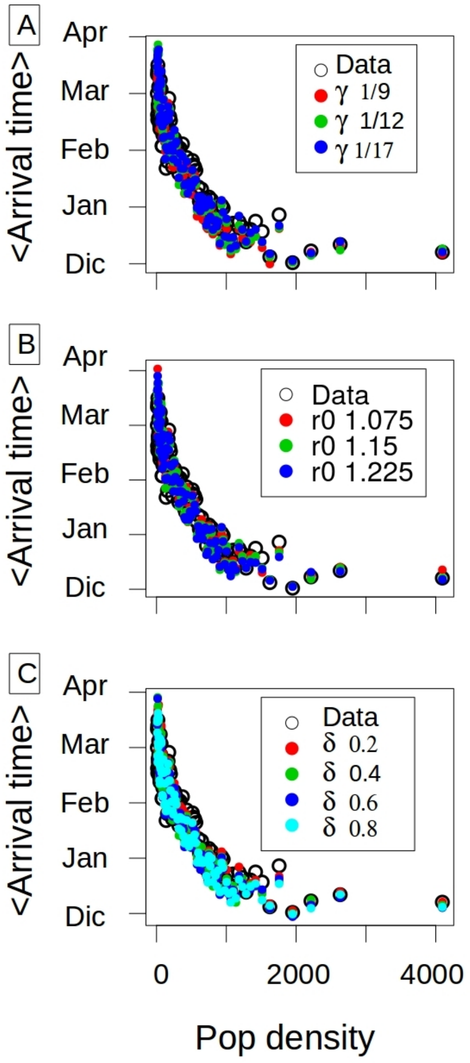

Supplementary Fig. 5. Mean arrival time vs population density: **(A)** for different values of γ (δ =0.2, R_0=1.15, ρ =0.5); **(B)** for different values of R_0 (δ =0.2, γ =1/17, ρ =0.5); and **(C)** for different values of δ (R_0=1.15, γ =1/17 and ρ =0.5).

Supplementary Figure 6

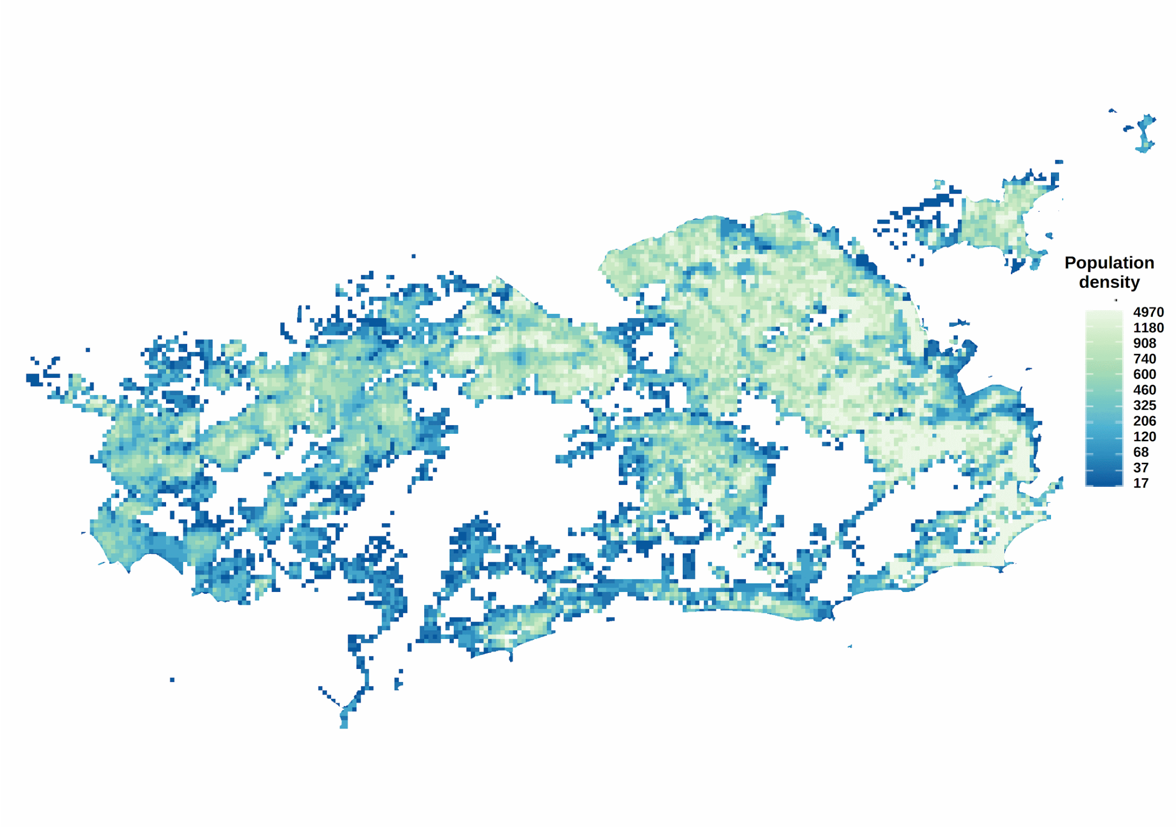

Supplementary Fig 6. Population density computed at the finest resolution of our units.

Supplementary Figure 7

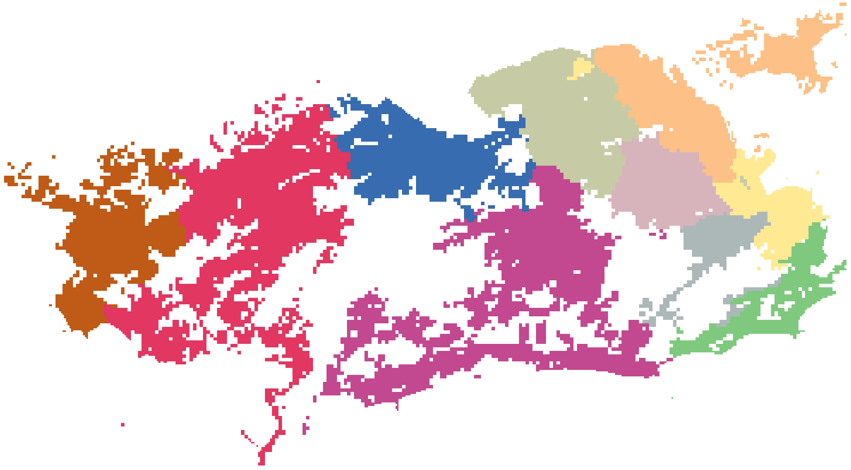

A)

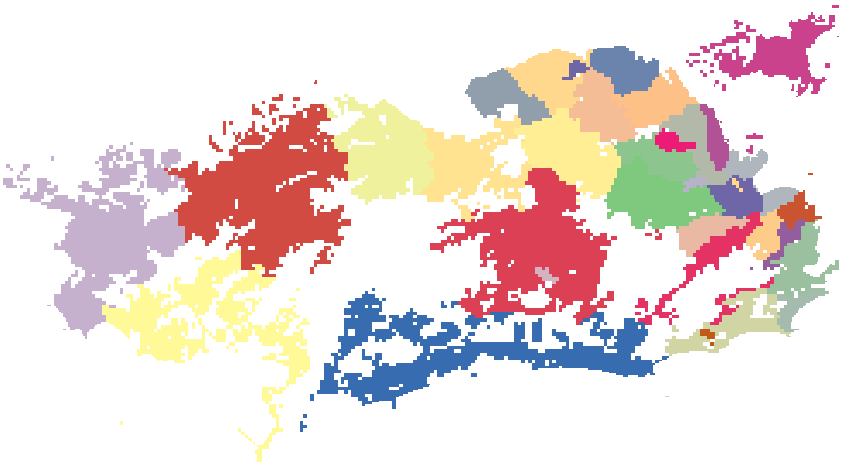

B)

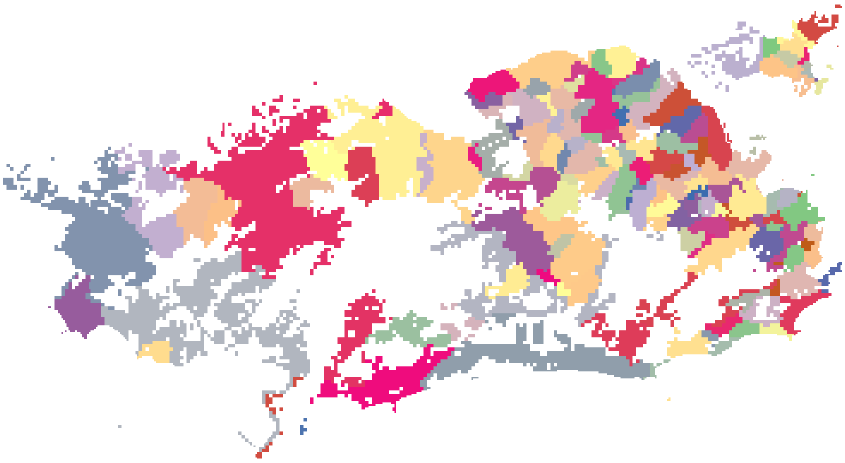

C)

Supplementary Fig. 7. Administrative partitions of the city of Rio de Janeiro: into (**A**) 10 regions, (**B**) 33 sub-regions and (**C**) 160 neighborhoods.

Supplementary Figure 8

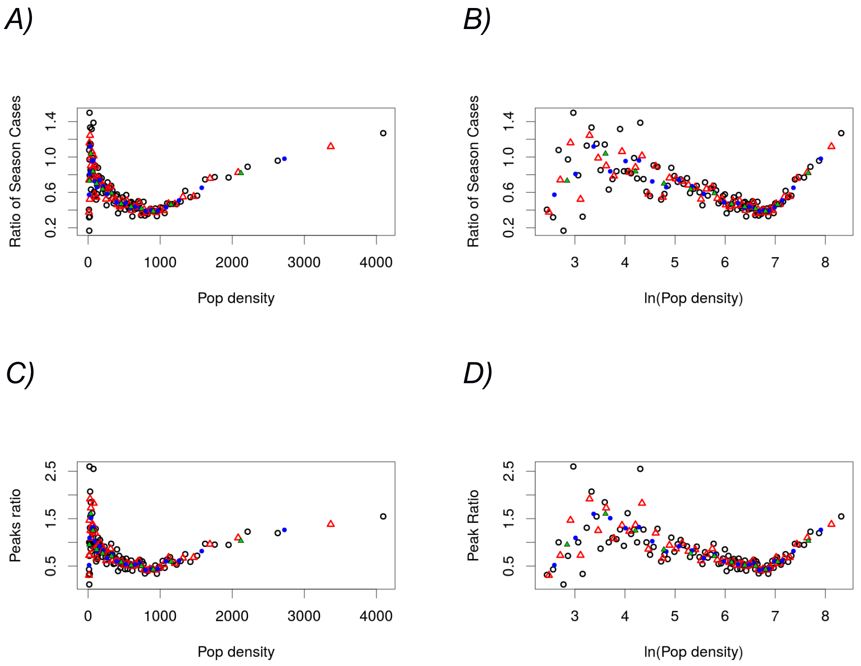

Supplementary Fig. 8. The upper row shows the ratio of cumulative cases in each season as a function of population density **(A)**, also in a log scale **(B)**. The bottom row displays the patterns obtained for the ratio of peak sizes in each season (regular and log scales are shown in **(C)** and **(D)** respectively).

Supplementary Note 1: HEALTHCARE CLINICS' DISTANCE

We obtained the locations of the public healthcare clinics in Rio de Janeiro city [https://www.data.rio/datasets/unidades-de-sa%C3%BAde-municipais/explore]. After excluding clinics that do not treat dengue cases, such as psychosocial care centers and referral centers, there were 302 public healthcare clinics in Rio de Janeiro. Supplementary Figure 9 shows the public healthcare clinics and the population per unit.

Supplementary Figure 9

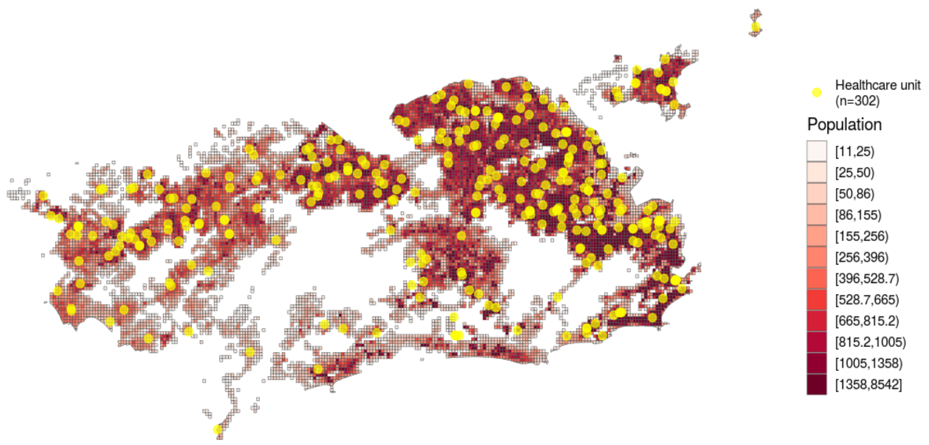

Supplementary Fig. 9. Map of the city showing the location of the public healthcare clinics (yellow dots) and the population density (population per unit –250 m by 250 m-).

Then, we calculated the euclidean distance between each grid unit's centroid to the nearest healthcare clinic. Supplementary Figure 10 shows population versus the distance to nearest public healthcare clinic. Roughly, most grid units have a distance of less than 2 km

Supplementary Figure 10

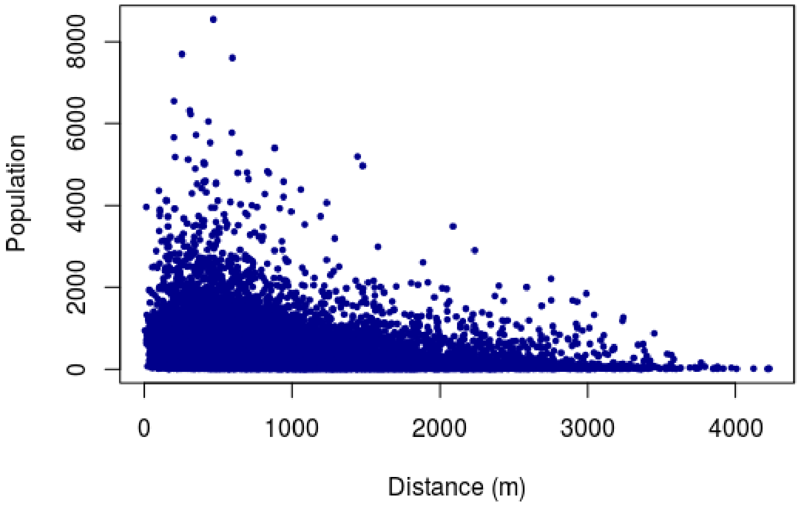

Supplementary Fig. 10. Unit population (population in 250 m by 250 m) as function of unit distance (in meters) to nearest public healthcare clinic. (The total number of units is 11247).

In fact, as Table S2 shows, for 95.6% of the grid units, the nearest healthcare clinic is less than 2.5 km away.

Distance to nearest public healthcare clinic (meters)	% of grid units
[0,500)	24.78
[500,1000)	35.98
[1000,2500)	34.80
[2500,3000)	2.92
[3000,3500)	1.21
[3500,4000)	0.27
[4000,4230]	0.04

Supplementary Table 2. Groups of distance to the nearest public healthcare clinic and the corresponding percent of units belonging to each group.

Finally, we analyzed the distribution of the distances to the nearest public healthcare unit by population density group shown on Supplementary Figure 11. As population density increases, there is a decreasing trend on the mean distance to the nearest public healthcare clinic, that goes from 1.6 km to 0.6 km. Despite this trend, the difference of the mean distances is very small, going from 1.6 to 0.6 km. A maximum increase of 1 km in distance should not impact the demand for medical assistance.

Supplementary Figure 11

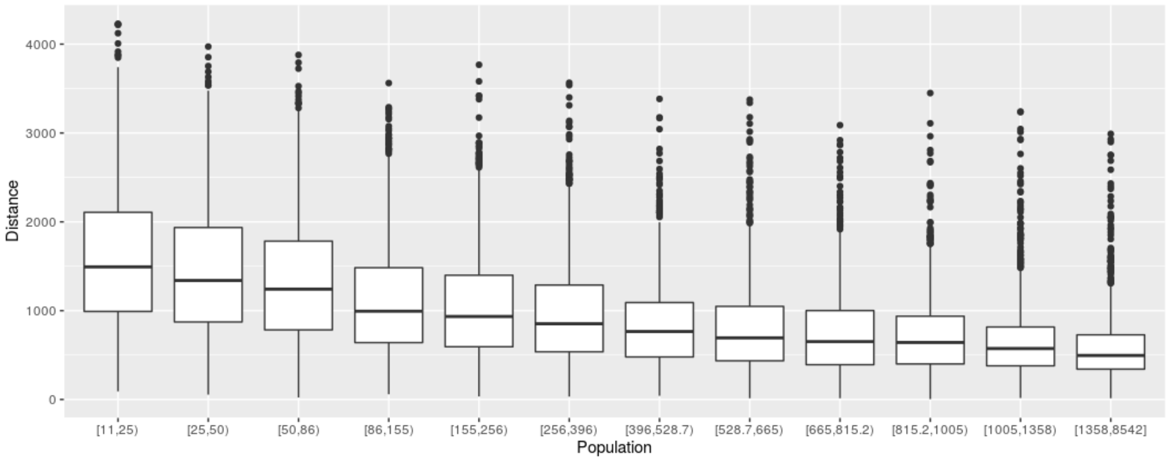

Supplementary Fig. 11. Box plot of the unit distance (meters) to the nearest public healthcare clinic as function of the unit population. The box plot is produced with the total number of units (11247 units, and each box has about 937 samples). The boxes represent the median with the 25th and 75th percentiles as is customary, the whiskers depict the extremes of the distribution without the outliers, and the points are the outliers (the values beyond Q3 + 1.5 IQR, where Q3 is the 75th percentile and IQR is the Interquartile Range).

Supplementary Note 2: COMPARISON OF THE ESTIMATED POPULATION WITH THAT FROM THE WorldPop DATASET

We chose to compare our estimates with the WorldPop dataset (2) because i) the estimates are also calculated based on Census data and are available for 2010, ii) the pixel size is of 100m, smaller than the size of our grid unit, and iii) it is open access. The WorldPop dataset contains the estimated total number of people per grid-cell at a resolution of 3 arc (approximately 100 m at the equator). The mapping approach is Random Forest-based dasymetric redistribution (3).

The total Rio de Janeiro's population was 6307639 according to the WorldPop dataset, and 6316636 in our grid. According to the National Census, there were 6320446 inhabitants in Rio de Janeiro city in 2010. Therefore, our total population is closer to the official one. To compare the estimates spatially, for each unit *u* of our grid (measuring 250x250m), with *u*= 1, 2, …, 20212, we calculated the population counts of the overlapping 100 m pixels from the WorldPop dataset. Supplementary Figure 9 shows that the populations are highly correlated, with R=0.94. The dashed line represents a 1 to 1 relationship. We can observe that the regression line (the solid line) is very close to the dashed line.

Supplementary Figure 12

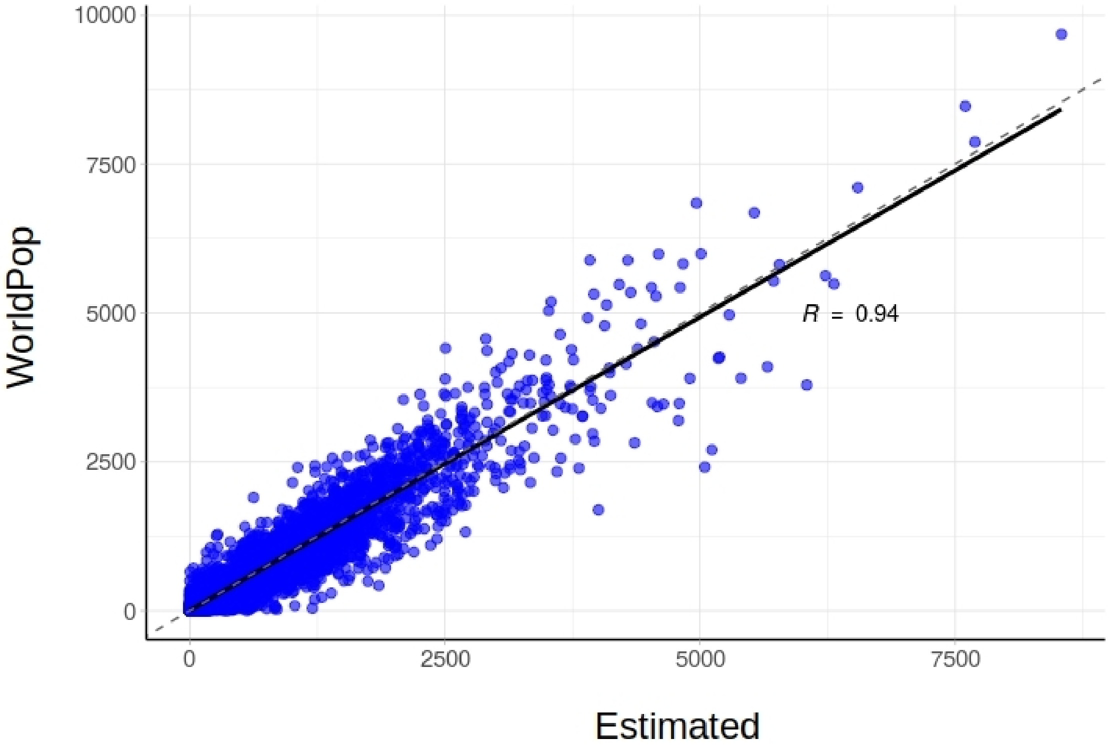

Supplementary Fig. 12. Comparing gridded population, Rio de Janeiro, Brazil 2010. Correlation of the population in 250² m² computed from WorldPop and that estimated in this article (See Population on the Grid subsection in main text).

Supplementary Figure 13

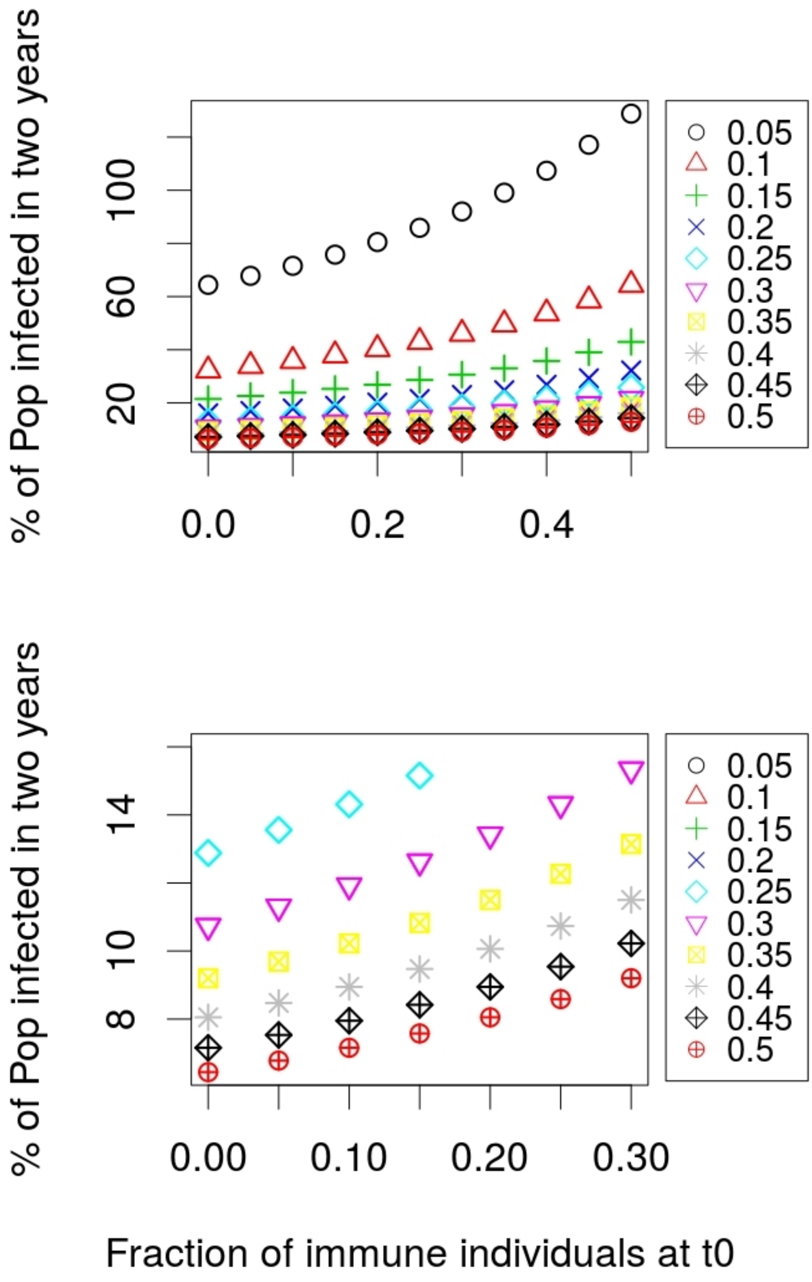

Supplementary Fig. 13. Percent of the total population infected in two seasons as a function of the initial fraction of the population immune at t_0, for different values of the reporting rate (indicated to the right of the plot with different symbols and colors). The bottom graph zooms in into the smallest range of initial fraction immune.

Supplementary Figure 14

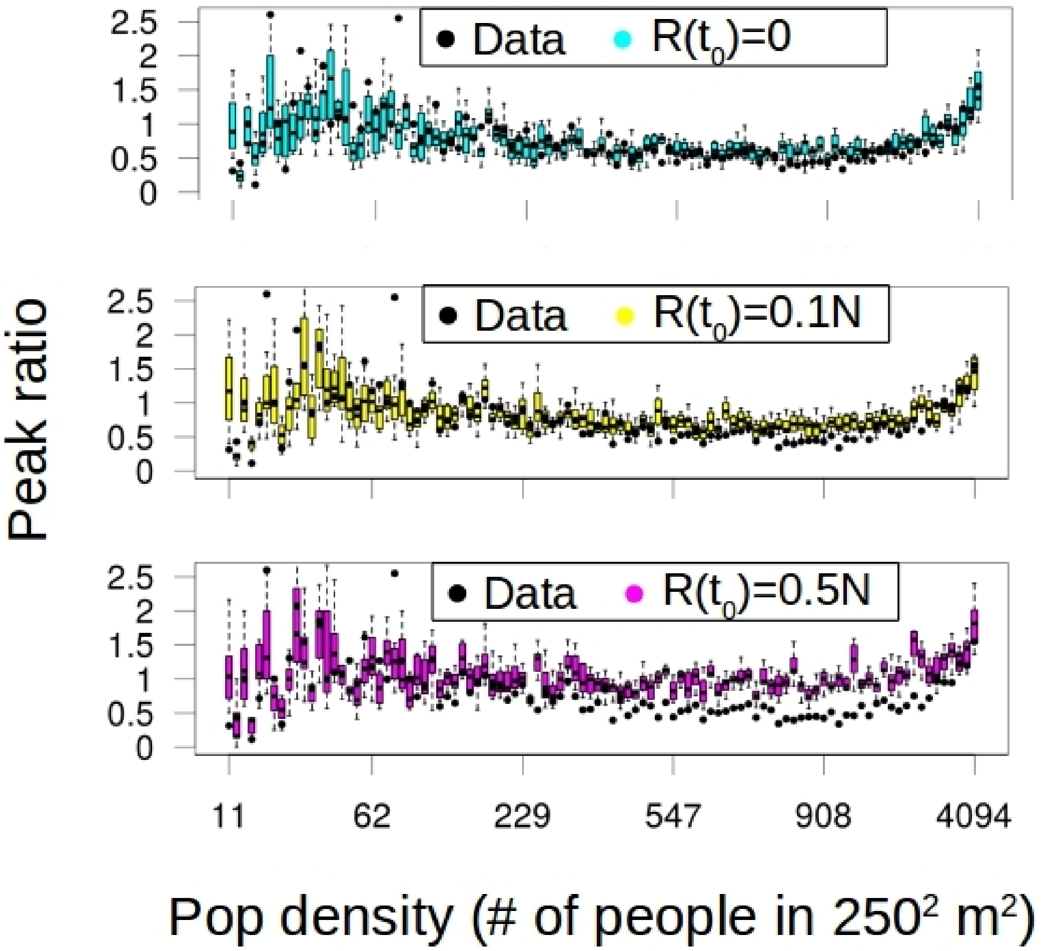

Supplementary Fig. 14. Peak ratio vs population density for different numbers of individuals in the initial recovered class (i.e. different number of initial susceptible individuals). Light blue, yellow and pink colors correspond respectively to 100%, 90% and 50% of the initial population susceptible to DENV4 (for a reporting rate of 0.5). The boxplots are computed from 20 stochastic realizations (in these, as is standard, the boxes represent the median with the 25th and 75th percentiles, the dotted lines depict the extremes of the distribution without outliers). For comparison, the empirical values of peak ratio are also shown (as black dots, for the 100 groups).

Supplementary Figure 15

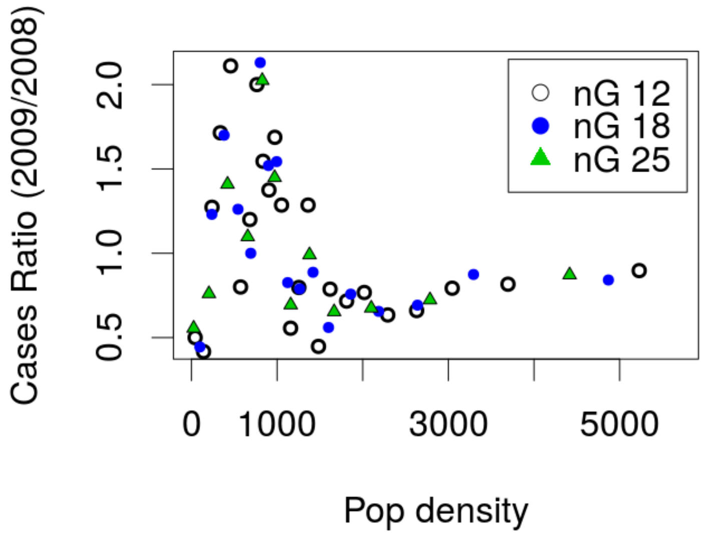

Supplementary Fig. 15. Ratio of the number of cases in seasons 2009 and 2008 (spatial resolution 250m by 250m) as a function of population density for the city of Delhi (India). Dataset from (*4*).

Supplementary References

1. R. Subramanian, V. Romeo-Aznar, E. Ionides, C. T. Codeço, M. Pascual, Predicting re-emergence times of dengue epidemics at low reproductive numbers: DENV1 in Rio de Janeiro, 1986–1990. *J. R. Soc. Interface*. **17**, 20200273 (2020).

2. Worldpop, WorldPop :: DOI: 10.5258/SOTON/WP00645, (available at https://www.worldpop.org/doi/10.5258/SOTON/WP00645).

3. F. R. Stevens, A. E. Gaughan, C. Linard, A. J. Tatem, Disaggregating census data for population mapping using random forests with remotely-sensed and ancillary data. *PLoS One*. **10**, e0107042 (2015).

4. O. Telle, A. Vaguet, N. K. Yadav, B. Lefebvre, E. Daudé, R. E. Paul, A. Cebeillac, B. N. Nagpal, The Spread of Dengue in an Endemic Urban Milieu–The Case of Delhi, India. *PLOS ONE*. **11** (2016), p. e0146539.
