# Supplementary material for: Fine-scale heterogeneity in population density predicts wave dynamics in dengue epidemics
Source: Nat Commun. 2022 Feb 22;13:996. doi: 10.1038/s41467-022-28231-w (PMC8864019; doi:10.1038/s41467-022-28231-w)
Supplement: Supplementary file 3 — Reporting Summary [file 41467_2022_28231_MOESM3_ESM.pdf]

nature research

Corresponding author(s): Mercedes Pascual

Last updated by author(s): 10/25/2021

Reporting Summary

Nature Research wishes to improve the reproducibility of the work that we publish. This form provides structure for consistency and transparency in reporting. For further information on Nature Research policies, see our Editorial Policies and the Editorial Policy Checklist.

Statistics

For all statistical analyses, confirm that the following items are present in the figure legend, table legend, main text, or Methods section.

n/a	Confirmed	
☐	☒	The exact sample size (n) for each experimental group/condition, given as a discrete number and unit of measurement
☐	☒	A statement on whether measurements were taken from distinct samples or whether the same sample was measured repeatedly
☒	☐	The statistical test(s) used AND whether they are one- or two-sided<br>*Only common tests should be described solely by name; describe more complex techniques in the Methods section.*
☐	☒	A description of all covariates tested
☐	☒	A description of any assumptions or corrections, such as tests of normality and adjustment for multiple comparisons
☐	☒	A full description of the statistical parameters including central tendency (e.g. means) or other basic estimates (e.g. regression coefficient) AND variation (e.g. standard deviation) or associated estimates of uncertainty (e.g. confidence intervals)
☒	☐	For null hypothesis testing, the test statistic (e.g. F, t, r) with confidence intervals, effect sizes, degrees of freedom and P value noted<br>*Give P values as exact values whenever suitable.*
☒	☐	For Bayesian analysis, information on the choice of priors and Markov chain Monte Carlo settings
☒	☐	For hierarchical and complex designs, identification of the appropriate level for tests and full reporting of outcomes
☐	☒	Estimates of effect sizes (e.g. Cohen's d, Pearson's r), indicating how they were calculated

Our web collection on statistics for biologists contains articles on many of the points above.

Software and code

Policy information about availability of computer code

Data collection	To create the grid and edit the census tract layer we used QGIS (version 3.6.3), and to obtain the population in the grid we used the R software with the packages tidyverse and sf.
Data analysis	The peak ratio analysis was performed in R. The stochastic SIR model was implemented in R with the package POMP. The deterministic SIR simulations were performed in C. The codes are available at https://github.com/vromeoaznar/DengueRio_peakRatio (under doi:10.5281/zenodo.5761972).

For manuscripts utilizing custom algorithms or software that are central to the research but not yet described in published literature, software must be made available to editors and reviewers. We strongly encourage code deposition in a community repository (e.g. GitHub). See the Nature Research guidelines for submitting code & software for further information.

Data

Policy information about availability of data

All manuscripts must include a data availability statement. This statement should provide the following information, where applicable:

- Accession codes, unique identifiers, or web links for publicly available datasets
- A list of figures that have associated raw data
- A description of any restrictions on data availability

The population data and time series of presence and absence of infections for each unit, as well as the aggregated time series of cases by administrative region and population density group, are available at https://github.com/vromeoaznar/DengueRio_peakRatio (under doi:10.5281/zenodo.5761972).. Requests concerning the raw epidemiological data should be made to the Secretariat of Health of Rio de Janeiro city.

Field-specific reporting

Please select the one below that is the best fit for your research. If you are not sure, read the appropriate sections before making your selection.

☐ Life sciences ☐ Behavioural & social sciences ☒ Ecological, evolutionary & environmental sciences

For a reference copy of the document with all sections, see nature.com/documents/nr-reporting-summary-flat.pdf

Ecological, evolutionary & environmental sciences study design

All studies must disclose on these points even when the disclosure is negative.

Study description	Using dengue surveillance data at high spatial resolution (250 m X 250 m) from Rio de Janeiro, we investigate the behavior of the size of successive epidemics with population density following DENV4 emergence. We rely on SIR models (both deterministic and stochastic) to understand the documented pattern and the associated interaction of local herd immunity with seasonal transmission.
Research sample	During the five years we analyze, (2010-2014), Rio de Janeiro experienced three major dengue outbreaks, dominated first by the DENV1 serotype, then followed for two consecutive years by the emergent DENV4 serotype, newly arrived in Brazil. We focus on DENV4 outbreaks (seasons 2011-2012 and 2012-2013) to assume that the whole population was initially susceptible to the virus.
Sampling strategy	Population dataset: The census tract areas for which the original dengue cases are reported, vary in size and can be bigger than a grid unit (250 mX250 m), especially in the least populated areas of the city. To generate the data set of dengue cases on the grid, we cropped from the census tract layer the areas classified as non-urbanized (such as water bodies, swamps, agricultural areas, green areas, beaches, rocky outcrops) in 2010 by the City Hall of Rio de Janeiro (layer available at <http://www.data.rio/datasets/uso-do-solo-2010>). The population of each census tract was then distributed randomly (uniformly) in the remaining areas obtained from deleting non-urban areas. The population within the units is computed by adding the grid layer.<br>Dengue cases were geocoded according to address of residency, and then counted for each grid unit by the Secretariat of Health of the city.
Data collection	Dengue Cases: Dengue is a disease of compulsory notification in Brazil, and cases are notified at the Sistema de Informação de Agravos de Notificação [Information System on Diseases of Compulsory Declaration] (SINAN)<br>Population: The population data is obtained from the Census 2010 (IBGE) (https://www.ibge.gov.br/estatisticas/downloads-estatisticas.html)
Timing and spatial scale	The dengue dataset corresponds to monthly cases between January 2010 and December 2014 , geocoded according to address of residency.<br>Population data is obtained from the Census 2010 at the census tract level.<br>Both datasets were scaled to a regular grid with units of 250 m by 250 m.
Data exclusions	We do not consider non-urbanized areas (such as water bodies, swamps, agricultural areas, green areas, beaches, rocky outcrops). We do not consider units with small effective areas and/or populations sizes less or equal than 10 in our analysis (as a result, we excluded 8954 units from 20212). Very small areas and population sizes are highly sensitive to the non-urban classification and the random distribution of the census tract population.
Reproducibility	For the deterministic simulations no reproducibility issue applies by definition. We specify the parameters, equations, and initial conditions for the model. We also consider a stochastic model (based on Poisson processes) to better address the empirical patterns. Here too results should be reproducible as long as sufficient realizations of the stochastic model are considered.
Randomization	We binned the units (250mX250 m) into G groups and aggregated their times series of reported cases. The groups were generated according to two aspects: 1) the geographical location of the units as determined by the administrative divisions of the city (10 areas, 33 regions, and 160 neighborhoods); and 2) the population of the units based on quantiles in order to obtain equal size groups and avoid statistical effects due to group size. We considered specifically four different partition levels, resulting in 12, 25, 50 and 100 groups with about 900, 450, 225, and 100 units respectively (from a total number of 11247 units for the whole city).
Blinding	Blinding is not applicable to our study. The data consist of reported cases in space and time. There is no trial and no blinding.

Did the study involve field work? ☐ Yes ☒ No

Reporting for specific materials, systems and methods

We require information from authors about some types of materials, experimental systems and methods used in many studies. Here, indicate whether each material, system or method listed is relevant to your study. If you are not sure if a list item applies to your research, read the appropriate section before selecting a response.

Materials & experimental systems

n/a	Involved in the study
☒	☐ Antibodies
☒	☐ Eukaryotic cell lines
☒	☐ Palaeontology and archaeology
☒	☐ Animals and other organisms
☒	☐ Human research participants
☐	☒ Clinical data
☒	☐ Dual use research of concern

Methods

n/a	Involved in the study
☒	☐ ChIP-seq
☒	☐ Flow cytometry
☒	☐ MRI-based neuroimaging

Clinical data

Policy information about clinical studies

All manuscripts should comply with the ICMJE guidelines for publication of clinical research and a completed CONSORT checklist must be included with all submissions.

Clinical trial registration	The data are not from a clinical trial
Study protocol	NA
Data collection	Dengue cases notified in Rio de Janeiro between January 2010 and December 2014 and geocoded according to address of residency
Outcomes	Dengue cases are geocoded according to address of residency, and then counted for each grid unit (250 m by 250 m) by the Secretariat of Health of the city. We (the co-authors) did not collect these data. We obtained them from the city and used them in our analysis.
